# Neoadjuvant Treatment in Rectal Cancer: Actual Status

**DOI:** 10.1155/2011/839742

**Published:** 2011-09-21

**Authors:** Ingrid Garajová, Stefania Di Girolamo, Francesco de Rosa, Jody Corbelli, Valentina Agostini, Guido Biasco, Giovanni Brandi

**Affiliations:** Department of Hematology and Oncology Sciences “L. A. Seragnoli”, Sant'Orsola-Malpighi Hospital, University of Bologna, via Massarenti 9, 40138 Bologna, Italy

## Abstract

Neoadjuvant (preoperative) concomitant chemoradiotherapy (CRT) has become a standard treatment of locally advanced rectal adenocarcinomas. The clinical stages II (cT3-4, N0, M0) and III (cT1-4, N+, M0) according to International Union Against Cancer (IUCC) are concerned. It can reduce tumor volume and subsequently lead to an increase in complete resections (R0 resections), shows less toxicity, and improves local control rate. The aim of this review is to summarize actual approaches, main problems, and discrepancies in the treatment of locally advanced rectal adenocarcinomas.

## 1. Indication and Benefit of Neoadjuvant Treatment

Rectal cancer is one of the most common cancers and accounts for approximately 1/3 of the deaths due to colorectal cancer in 2009 [1]. In well-selected patients (i.e., those with well-differentiated T1 cancers involving <40% of the circumference, without lymphovascular invasion), particularly when the only other option is abdominoperineal resection (APR), local excision seems to be a viable option [2]. Locally advanced rectal cancer is comprised of tumors with extension beyond the muscularis propria (≥T3) and/or those with clinical or pathologic evidence for lymph node metastasis (N+); in these cases multimodality approaches are recommended [1]. Such multimodality approaches are applicable to patients with rectal cancers at or below the peritoneal reflection. This designation generally represents cancers below 12 cm from the anal verge. Generally, the treatment of tumors localized more than 12 cm from anal verge is based on the colon cancer paradigm. The determination of “node positivity” in patients with locally advanced rectal cancer can be difficult. Most lymph nodes involved by rectal cancer are less than 1 cm, but not all lymph nodes detected by MRI or TRUS represent metastatic disease; therefore, some patients can be understaged. Neoadjuvant CRT may also be considered if the preoperative staging evaluation suggests the presence of mesorectal invasion [3]. This finding is highly predictive of residual tumor at the circumferential margin [4]. 

Neoadjuvant CRT is more effective than adjuvant therapy in reducing local recurrence and in minimizing toxicity [5]. It is associated with tumor downstaging, significantly higher rate of pathologic complete response (pCR), significantly less advanced pT and pN stage, and fewer cases with venous, perineural, or lymphatic invasion, increased tumor resectability [6]. Multivariate analyses confirmed that the response to neoadjuvant CRT was predictive of improved OS among the patients with locally advanced rectal cancer [7, 8]. Taking advantage of tumor downstaging after neoadjuvant CRT is supposed to increase the chance of sphincter saving surgery (SSS) [9]. In fact, this hypothesis is a very complex issue involving the stage and location of the tumor, the patient habitus and desire, and the skill of the surgeon. In a very recent review [9], a total of 17-randomized trials were analysed, to answer the question if neoadjuvant treatment in rectal cancer is able to increase SSS. The authors concluded that the analysis of the most recent and large phase III trials does not support this hypothesis. The obvious reduction of permanent stoma in the recent years is according the authors mainly due to technical and conceptual improvements in the surgical management of rectal cancers [9]. No data concerning disease progression during treatment were reported in the large phase III neoadjuvant trials [5, 10, 11].

## 2. Short- versus Long-Course Neoadjuvant Radiation

There are two types of neoadjuvant radiation regimens accepted as standard for resectable rectal cancer: short-course (5 × 5 Gy) RT alone with immediate surgery and long-course combined CRT with delayed surgery (conventional radiation doses of 1.8–2 Gy per fraction over 5-6 weeks, for a total dose of 45–50.4 Gy) [5]. 

The Polish trial [12] compared neoadjuvant short-course RT followed by total mesorectal excision (TME) within 7 days and neoadjuvant long-course CRT followed by TME at 4–6 weeks. Neoadjuvant short-course RT had less grade 3/4 acute toxicity (3.2 versus 18%), better compliance (97.9 versus 69.2%), and similar postoperative toxicity (28.3 versus 27%). Neoadjuvant long-course CRT had higher rate of pCR (16.1 versus 0.7%) and lower circumferential margin involvement (4.4 versus 12.9%), which did not translate into improved survival or recurrence at a median follow up of 48 months. 98% of the patients receiving short-course RT completed prescribed treatment compared with only 69.2% of patients receiving CRT [13]. 

The optimal time interval between RT and surgery is unknown. There is a trend towards greater downstaging and complete response with increasing interval between long-course CRT and surgery [14]. The Lyon 90-01 trial [15] randomized patients with T2-3 mid- and low-rectal cancer to neoadjuvant 13 × 3 Gy RT (not short-course RT) followed by short interval (within 2 weeks) or long interval (6–8 weeks) to surgery. Long interval arm demonstrated better tumor response (72 versus 53%) and pathological downstaging (26 versus 10%). There was a trend towards increased sphincter preservation in the long-interval group (76 versus 68%) which was not statistically significant. There was no significant difference in 3-year OS (81 versus 73%), local recurrence (10.3 versus 9.9%), early postoperative mortality (3 versus 4%), or morbidity (18 versus 17%). In another multicenter trial, patients were randomized to either short-course RT (5 × 5 Gy) and surgery within 1 week, short-course RT and surgery after 4–8 weeks or long-course RT (25 × 2 Gy) and surgery after 4–8 weeks. Compliance was acceptable and severe acute toxicity was low, irrespective of fractionation. The patients receiving short-course RT with surgery 11–17 days after the start of RT had the highest complication rate. These results indicate that surgery should be performed immediately after short-course RT, within approximately 5 days after the last RT fraction, or be delayed for more than 4 weeks [16, 17]. A nonsignificant improvement in SSS was reported in a French study which randomized patients to surgery within two weeks after the completion of RT, compared with six to eight weeks. The long interval between neoadjuvant RT and surgery provided increased tumor downstaging with no detrimental effect on toxicity but did not result in significant differences in long-term local control or survival [18, 19]. The small number of fractions makes short-course RT less expensive and more convenient than CRT. The optimal fractionation of RT, timing of surgery, and the best use of concomitant CT remain controversial.

## 3. Choice of Chemotherapy Regimen

For patients with clinical stage II and III rectal cancer, neoadjuvant treatment with RT and 5-FU-based CT is recommended. The integration of newer chemotherapeutic and targeted agents in patients with advanced colorectal cancer have led to further improvements in DFS and OS. These agents are now being studied with RT in the neoadjuvant therapy of rectal cancer.

Infusional versus bolus 5-FU: infusional rather than bolus 5-FU during RT increases the likelihood of a pCR in patients with locally advanced rectal cancer [20]. 

Orally active fluoropyrimidines versus infusional 5-FU: recently, results of NSABP R-04 trial have been published. In the study, 1680 patients were randomly assigned to the four treatment groups: continuous intravenous infusion of 5-FU (225 mg/m² 5 days per week) with or without oxaliplatin (5 cycles of 50 mg/m² weekly), or capecitabine (1650 mg/m² 5 days per week) with or without oxaliplatin. The authors conclude that the administration of capecitabine with neoadjuvant RT achieved rates similar to continuous infusion 5-FU for surgical downstaging, SSS, and pCR. Further results of the study, including DFS and OS, should be available by fall 2013 [21]. In another phase III trial comparing capecitabine with 5-FU for adjuvant or neoadjuvant CRT for locally advanced rectal cancer [22], results demonstrated that capecitabine may replace 5-FU in the perioperative treatment of locally advanced rectal cancer because of the advantageous safety profile, improved nodal downstaging, and more favorable survival outcomes seen in the study. Overall, 392 patients were randomly assigned, 197 to the capecitabine group and 195 to the 5-FU group. Of these groups, 81 patients received capecitabine as neoadjuvant therapy and 80 received 5-FU in addition to TME. Hand-and-foot syndrome (HFS), fatigue, and proctitis were more commonly observed in patients in the capecitabine group, while leucopenia and alopecia were more frequent in the 5-FU group. Rates of diarrhea were similar in patients in the capecitabine and 5-FU groups during cycles where no radiation was given, but significantly more diarrhea was noted in patients given capecitabine at the same time as RT compared with patients administered 5-FU and RT (*P* = 0.07). After 52 months of follow up, local recurrence rates were equal in the two arms (6.1% capecitabine and 7.2% 5-FU; *P* = 0.7795), but fewer patients in the capecitabine group developed distant metastases (18.8% versus 27.7%; *P* = 0.0367). Fifty five of 93-reported deaths occurred in patients in the 5-FU arm. Analysis showed that capecitabine was noninferior compared with 5-FU in 5-year OS, which was the study's primary endpoint (75.7% versus 66.6%, respectively; *P* = 0.0004). 3-year DFS was superior in the capecitabine group (75.2%) compared with the 5-FU group (66.6%; *P* = 0.034). Development of HFS predicted more favorable outcomes. In the study, any patient who developed the skin disorder had significantly higher 3-year DFS (83.2%; *P* = 0.004) and 5-year OS (91.4%; *P* < 0.0001) compared with patients who did not develop HFS.

Leucovorin: in a retrospective review of 297 patients with locally advanced rectal cancer treated with 5-FU-based CT and concurrent RT, the most common CT protocol was 5-FU bolus with leucovorin, both given for five days on weeks 1 and 5 of RT. The pCR rate was 15% [23]. In one report of 22 patients with locally advanced rectal cancer treated using infusional 5-FU combined with leucovorin concurrent with RT, the pCR rate was 14%. 82% of patients had a SSS, and 3-year survival rate was 69% [24]. The role of this drug in rectal cancer is not so clear.

Oxaliplatin: two-phase III randomized studies have compared the addition of oxaliplatin to either 5-FU (STAR-01) or capecitabine (ACCORD 12/0405) [25, 26]. The addition of weekly oxaliplatin significantly increased toxicity without improving the pCR or SSS. Originally designed as a two-arm study comparing capecitabine with 5-FU, oxaliplatin was added to the study protocol (known as NSABP R-04). Although, oxaliplatin has theoretical advantages as a radiosensitizing agent, no difference was noted in patients receiving capecitabine with or without oxaliplatin or 5-FU with or without oxaliplatin, but greater toxicity was noted in both oxaliplatin groups [21]. In conclusion, the addition of oxaliplatin to fluoropyrimidine-based CRT cannot be considered a standard approach in patients with locally advanced rectal cancer. Oxaliplatin during RT should not be used outside clinical trial.

Irinotecan: benefit could not be shown neither for the addition of irinotecan to 5-FU in patients with locally advanced rectal cancer in neoadjuvant setting. 106 patients with locally advanced rectal cancer were randomly assigned to continuous infusional 5-FU concurrent with hyperfractionated RT or to infusional 5-FU plus irinotecan and concurrent conventional fractionation RT [27]. The pCR rate was similar in both arms. In recently published results of the phase II trial [28], the rate of pCR of MRI-defined locally advanced rectal adenocarcinoma using concurrent irinotecan and capecitabine was 22%. A Radiation Therapy Oncology Group randomized study compared oxaliplatin and capecitabine with RT versus irinotecan and capecitabine with RT. The pCR rates were 18% in the oxaliplatin arm and 10% in the irinotecan arm [29]. Irinotecan has not benefit in clinical response and moreover increase treatment-related toxicities.

EGFR inhibitors: overexpression of EGFR is regarded as a negative prognostic factor and is associated with resistance to RT. In retrospective analyses, patients with EGFR-expressing rectal cancer undergoing neoadjuvant RT had a significantly lower DFS and lower chance of achieving a pCR [30–36]. Two types of EGFR inhibitors have been tested in patients with locally advanced rectal cancer in neoadjuvant setting: small-molecule EGFR tyrosine kinase inhibitor (gefitinib) and monoclonal antibody to EGFR (cetuximab).

Gefitinib: in a phase I trial from Duke University combining gefitinib, capecitabine, and RT in rectal cancer, the combination resulted in significant toxicity, and no recommended phase II dose could be determined [37]. In contrast, an Italian study evaluating infusional 5-FU with gefitinib and RT showed good tolerability with a pCR rate of 30.3% [38]. Ongoing phase I to II studies are further evaluating the tolerability and efficacy of gefitinib with conventional neoadjuvant CRT regimens in patients with locally advanced rectal cancer.

Cetuximab: cetuximab can be safely combined with RT and CT in the neoadjuvant treatment of rectal cancer. Two phase II studies investigating cetuximab delivered with oxaliplatin/capecitabine-based [39] and 5-FU-based [40] CRT yielded disappointing pCR rates of only 9% and 5%, respectively. Another study [41] suggests EGF A + 61G polymorphism to be a predictive marker for pCR, independent of KRAS mutation status, to cetuximab-based neoadjuvant CRT of patients with locally advanced rectal cancer. 130 patients with locally advanced rectal cancer who were enrolled in phase I/II clinical trials treated with cetuximab-based CRT were included. Patients with the EGF 61 G/G genotype had pCR of 45%, compared with 21% in patients heterozygous, and 2% in patients homozygous for the A/A allele (*P* < 0.001). 

Antiangiogenic agents: increased levels of vascular endothelial growth factor (VEGF) expression have been found in the tumors and sera of patients with localized as well as metastatic colon and rectal cancer [42–44]. High VEGF expression has been associated with disease progression and inferior survival. 

Bevacizumab: the addition of bevacizumab to 5-FU-based CRT provides encouraging pCR rates and does not increase acute toxicity [45, 46]. Plasma VEGF (vascular endothelial growth factor), PlGF (placental-derived growth factor), sVEGFR1 (soluble vascular endothelial growth factor receptor), and IL-6 and CECs (circulating endothelial cells) should be further evaluated as candidate biomarkers of response for this regimen [45]. However, the impact of this strategy on long-term outcomes and posttreatment complications awaits the completion of phase III studies.

## 4. Induction Chemotherapy before Neoadjuvant Chemoradiotherapy in Patients with Locally Advanced Rectal Cancer

Multimodality treatment for patients with locally advanced rectal cancer include radiotherapy, chemotherapy, chemoradiotherapy, surgery, and eventual incorporation of molecularly targeted agents. The optimum sequence of these modalities is being discussed. Moreover, rectal cancer local recurrence rates are today less than 10%. The predominant mode of failure in rectal cancer is the development of distant metastases (30–35%). Therefore, the primary goal of adding induction CT is not to improve local efficacy, but to better control distant disease [5, 47]. The neoadjuvant RT/CRT has been shown to be superior to adjuvant treatment for variety of endpoints [5, 48]. Another consideration to underline is the suboptimal compliance of systemic treatment in adjuvant setting. Approximately 50% of patients are unable to receive the planned adjuvant CT dose [5, 10, 11, 49, 50]. Two common reasons for this are toxicity and patient refusal. Given the fact that the cumulative doses of the new drugs reached during neoadjuvant RT are substantially lower than in adjuvant colon cancer trials, an innovative approach such as to deliver systemic therapy prior to neoadjuvant CRT was developed [5, 10, 11, 49, 50]. Induction CT may be associated with better treatment compliance and may enable full systemic doses of CT to be delivered. Other theoretical advantages of induction CT include the possibility of tumor shrinking or downstaging, thereby facilitating more effective local treatment and early treatment of micrometastasis. Tumor shrinkage potentially allows improved tumor vascularity. Theoretically, the consequences of this are improved oxygenation and higher intratumoral concentration of cytotoxic drugs [51, 52]. Another theoretical advantage of induction CT is the potential to eradicate distant micrometastases at an early stage in the evolution, utilization of the embryonic tumor blood supply (in contrast to surgical scars), and treatment of fit patient before surgery [53]. On the other hand, this strategy may be associated with the selection of radioresistant clones, the induction of accelerated repopulation, possibly reduced compliance to CRT, and a substantial delay of definitive surgery [53]. 

Recently, a Spanish randomized phase II trial compared the induction CT approach with conventional neoadjuvant CRT followed by surgery and adjuvant CT. A total of 108 patients with locally advanced resectable rectal adenocarcinoma were randomly assigned to: arm A: preoperative CRT (5 weeks, capecitabine 1650 mg/m² 5 days/week + oxaliplatin 50 mg/m² weekly, pelvic RT: 50.4 Gy), followed by surgery after 5-6 weeks and adjuvant CRT CAPOX (4 cycles, capecitabine 2000 mg/m^2^ for 14 days, oxaliplatin 130 mg/m^2^ day 1). Arm B: induction CAPOX (4 cycles, capecitabine 2000 mg/m^2^ for 14 days, oxaliplatin 130 mg/m^2^ day 1) followed by CRT (5 weeks, capecitabine 1650 mg/m^2^ 5 days/week + oxaliplatin 50 mg/m^2^ weekly, pelvic RT: 50.4 Gy), followed by surgery after 5-6 weeks. The primary endpoint was pCR. Compared with adjuvant CAPOX, induction CAPOX before CRT had similar pCR and complete resection rates. It did achieve more favorable compliance and toxicity profiles [54]. Chau et al. published clinical results of phase II trials (first published in 2006 with 77 patients updated in 2010 with 105 patients). He examined the use of 4 cycles of induction CT CAPOX (oxaliplatin 130 mg/m^2^ day 1 with capecitabine 2000 mg/m^2^ daily for 14 days every 3 weeks) followed by CRT (54 Gy over 6 weeks with capecitabine 1650 mg/m^2^ daily), followed by total mesorectal excision, and 12 weeks of adjuvant capecitabine (2500 mg/m^2^ daily for 14 days every 3 weeks). The primary endpoint was pCR. Radiological response rates after induction CT and CRT were 74% and 89%, respectively. 3-year progression-free and overall survival were 68% and 83%, respectively. A 20 % pCR was reported. A major concern of this study is that nine patients had cardiac and thromboembolic toxic effects, leading to four deaths during induction CT; however, other trials with induction CT did not confirm such a high number of early fatal events [55]. Another phase II study evaluated the efficacy and safety of neoadjuvant capecitabine plus oxaliplatin and RT in patients with locally advanced rectal cancer. Treatment consisted of one cycle of XELOX (capecitabine 1000 mg m^2^ for 14 days and oxaliplatin 130 mg m^2^ day 1), followed by RT (1.8 Gy fractions 5 days per week for 5 weeks) plus CAPOX (capecitabine 825 mg m^2^ on days 22–35 and 43–56, and oxaliplatin 50 mg m² on days 22, 29, 43, and 50). Surgery was recommended 5 weeks after the completion of CRT. The primary endpoint was pCR. Sixty patients were enrolled. The pCR rate was 23%, and R0 resection was achieved in 98% of patients. Sphincter preservation was achieved in 84% of patients. Tumor and/or nodal downstaging was observed in 65% of patients. The most common grade 3/4 adverse events were diarrhea (20%) and lymphocytopaenia (43%) [56]. Another Spanish study compared efficacy in terms of pathologic response in patients with locally advanced rectal cancer treated with neoadjuvant CRT, with or without a short-intense course of induction oxaliplatin. 114 patients were treated with neoadjuvant CRT (45–50.4 Gy + oral Tegafur 1200 mg/day). 52 patients additionally received induction FOLFOX-4 (2 cycles), followed by the previously described Tegafur CRT regime. Surgery was performed in 5-6 weeks. Incidence of pT(0) specimens was significantly increased by induction FOLFOX-4 (*P* = 0.006). Total T and N downstaging was 58% versus 75% and 42% versus 40%, respectively (*P* = ns). T downstaging of > or = 2 categories was significantly superior in FOLFOX-4 group (*P* = 0.029). The authors conclude that short-intense induction FOLFOX-4 significantly improves pCR in patients with locally advanced rectal cancer treated with tegafur-sensitized neoadjuvant CRT [57]. 

In conclusion, the induction CT in patients with locally advanced rectal cancer is feasible, does not compromise CRT or surgical resection, and enables CT to be delivered in adequate dose and intensity. A phase III study to definitively test the induction strategy is warranted.

## 5. The Prognostic Impact of Tumor Regression

Modern neoadjuvant concurrent CRT treatment regimens have consistently demonstrated pCR rates of up to 20% [58, 59]. The Gastro-Intestinal Working Group of the Italian Association of Radiation Oncology collected clinical data for 566 patients with pCR after neoadjuvant therapy. Locoregional recurrence occurred in 7 patients (1.6%) and distant metastases in 49 patients (8.9%). Overall, 5-year rates of DFS and OS were 85% and 90%, respectively. These data confirm that in a large series of patients achieving pCR after neoadjuvant therapy, highly favorable outcomes are achieved [60]. Investigators from Memorial Sloan Kettering Cancer Center reported that response to neoadjuvant therapy was a strong predictor of DFS. However, outcome was most accurately estimated by final pathologic stage, which is influenced by both preoperative stage and response to therapy [61]. These results indicate that pathologic stage is still the most reliable predictor of survival in patients undergoing neoadjuvant CRT and surgery. The pCR appears to be associated with a very favorable prognosis. The degree of regression has been correlated to long-term survival outcomes. In a subgroup of patients from the German Rectal Cancer Trial, 5-year DFS ranged from 86% for patients with complete tumor regression, to 75% for patients with 25–75% tumor regression, and 63% for patients with <25% tumor regression (*P* = 0.006) [62]. Furthermore, OS was improved with downstaging (*P* = 0.003), and the persistence of positive lymph node involvement after treatment was strongly associated with a higher risk of recurrence (*P* < 0.001) [63]. Recognizing the prognostic importance of tumor regression, the Tumor Regression Grade (TRG) classification became an essential component of the protocol for pathologic reporting of rectal cancer resection specimens.

There is a question whether selected patients with radiologic and clinical evidence of a complete response after neoadjuvant CRT might be able to avoid surgery. No randomized trials are available. As it is sure that some patients with clinical complete response have microscopic residual tumor at resection, at the present, surgery remains the standard approach after neoadjuvant CRT, even in patients who appear to have a complete clinical response to neoadjuvant therapy.

## 6. Adjuvant Chemotherapy after Neoadjuvant Therapy for Rectal Cancer?

There is insufficient evidence on the benefit of adjuvant CT after neoadjuvant CRT for rectal cancer. The main advantage of adjuvant CT is better selection of patients since it can be based on pathologic staging [64]. In addition, the high rate of failure in terms of distant metastases suggests that residual malignant cells either in the primary site or elsewhere may require more effective and additional systemic methods of elimination [53]. The primary disadvantages include increased toxicity [64] which may compromise the dose intensity of adjuvant CT [53], and the compliance of adjuvant CT is suboptimal [10, 11].

The benefit of 5-FU-based adjuvant CT in patients with locally advanced rectal cancer undergoing neoadjuvant CRT remains uncertain, although most oncologists recommend it. NCCN guidelines recommend that all such patients should receive adjuvant CT even if they have a pCR after neoadjuvant therapy [65]. The choice of regimen is unsettled. ESMO guidelines stated that “similar to the situation in colon cancer stages III (and “high-risk” stage II), adjuvant CT can be provided, even if the scientific support for sufficient effect is less.” It appears as if the efficacy of adjuvant CT is less if the tumors have not responded to the neoadjuvant RT/CRT [66]. Experts of European Rectal Cancer Conference (EURECA-CC2) acknowledged that there is an insufficient evidence on the benefit of adjuvant CT after neoadjuvant CRT to come to consensus about its use [67]. Subgroup analysis suggests that only patients who respond and are downstaged from cT3-4 to ypT0-2 benefit from 5-FU-based adjuvant CT [68]. Survival benefit of 3-4% with 5-FU-based adjuvant CT was reported [69]. EORTC 22921 randomised trial failed to confirm a benefit of adjuvant CT in patients with locally advanced rectal cancer after neoadjuvant CRT in the terms of DFS or OS [11], even in the node-positive patients [68]. Data of 785 of the 1,011 randomly assigned patients whose disease was M0 at curative surgery were used. Although there was no statistically significant impact of adjuvant CT on DFS for the whole group (*P* > 0.5), the treatment effect differed significantly between the ypT0-2 and the ypT3-4 patients, only the ypT0-2 patients seemed to benefit from adjuvant CT (*P* = 0.011). The same pattern was observed for OS. Exploratory analyses suggest that only good-prognosis patients (ypT0-2) benefit from adjuvant CT. Patients in whom no downstaging was achieved did not benefit. This also suggests that the same prognostic factors may drive both tumor sensitivity for the primary treatment and long-term clinical benefit from further adjuvant CT [68]. Also Janjan et al. [8] suggested that patients who responded to 5-FU during neoadjuvant CRT probably would also respond to 5-FU-based adjuvant CT. It has been frequently observed that a large number of patients who remain in a node-positive stage after neoadjuvant CRT and surgery for rectal cancer developed early distant metastases and not local recurrence despite of continuing adjuvant CT [70]. Patients downstaged to ypT0-2N0 disease after CRT or after RT alone have a favourable prognosis [60, 71–73]. For these reasons, according to some authors, the gain in absolute percentages from adjuvant CT in those groups is very small [74]. It was reported that adding adjuvant CT did not significantly improve DFS or OS for patients with a good response (ypT0-2N0) following neoadjuvant CRT and curative surgery [75]. These findings are consistent with suggestion by Das et al. [76] that adjuvant CT may be of greater benefit for high-risk patients. Bujko et al. underlined some interesting consideration about EORTC 22921 trial [74]. The first point was that the intention-to-treat principle was not followed as 22% patients were excluded from the analysis. Furthermore, the numbers of patients in whom the benefit of CT was found (ypT0-2 disease) were imbalanced in the adjuvant CT group versus the control group (198 versus 225, respectively). Also, the beneficial effect of adjuvant CT was confined only to ypT0-2 patients receiving conventionally fractionated neoadjuvant RT and not those receiving neoadjuvant CRT [77]. A pooled retrospective analysis reported 566 patients with advanced rectal cancer with pCR after neoadjuvant RT or CRT [60]. Unexpectedly, a tendency towards worse DFS was noted in the 22% of patients given adjuvant CT, compared with those not receiving this treatment. Therefore, the concept of the EORTC trial subgroup analysis that adjuvant CT provides a benefit in patients downstaged after RT/CRT is dubious [74]. The aim of the QUASAR trial [69] was to determine survival benefit from adjuvant CT for patients with colorectal cancer at low risk of recurrence, for whom the indication for such treatment is unclear. After apparently curative resections of colon or rectal cancer, 3239 patients (2963 with stage II disease, 2291 with colon cancer), were randomly assigned to receive CT with 5-FU and folinic acid (*n* = 1622) or to observation (*n* = 1617). The primary outcome was all-cause mortality. After a median follow up of 5.5 years, there were 311 deaths in the CT group and 370 in the observation group; the relative risk of death from any cause with CT versus observation alone was 0.82 (95% CI 0.70–0.95; *P* = 0.008). There were 293 recurrences in the CT group and 359 in the observation group; the relative risk of recurrence with CT versus observation alone was 0.78 (0.67–0.91; *P* = 0.001). The authors concluded that CT with 5-FU and folinic acid could improve the survival of patients with stage II colorectal cancer although the absolute improvements are small (an absolute improvement in survival of 3.6%) [74].

Another clinically relevant question is whether the indication for adjuvant CT should be determined by clinical staging (cTNM) or by the definitive pathological surgical staging (ypTNM). Several reports have shown that the postoperative pathological staging after CRT is more discriminative for prognosis than the pretreatment clinical staging [60, 61, 72]. 

The role of adjuvant CT following neoadjuvant CRT and radical surgery for patients with locally advanced rectal cancer remains unclear. Randomized trials are needed.

## 7. The Problem of Low-Risk T3N0M0 Rectal Tumors

A number of studies [78–80] have demonstrated that patients undergoing resection of pT3N0 rectal cancer with favorable pathologic features experience a low rate of local failure after surgery alone, suggesting that these patients may not require adjuvant therapy. A ten-year actuarial local recurrence rate of these patients is less than 10% [81]. Gunderson et al. reported a retrospective analysis of pooled data demonstrating similar 5-year OS for pT3N0 rectal cancer patients treated with surgery and CT alone (84%) versus those treated with CRT (74% to 80%), further suggesting that trimodality therapy may be excessive for some patients in the T3N0 subset [82]. In the study of Lombardi et al., the authors found that 28% of patients with rectal cancer clinically staged as cT3N0 before CRT were identified to have lymph node metastases at surgical pathology. In the large multicenter study, 22% of patients staged before neoadjuvant CRT as having cT3N0 rectal cancer on either EUS or MRI have pathologically positive lymph nodes [83]. This subset of patients differs from the remaining true node-negative patients [84]. In meta-analysis of 90 imaging studies that aimed to compare EUS, CT, and MRI performance for rectal cancer staging were found low sensitivity values for nodal staging without statistically significant differences between each modality. Sensitivity estimates for EUS, CT, and MRI were 67, 55, and 66%, respectively. Specificity values were also comparable: 78% for EUS, 74% for CT, and 76% for MRI [85]. The German study [5] demonstrated that 18% of patients staged clinically as having cT3, cT4, or node-positive rectal cancer on EUS were overstaged. Because neoadjuvant CRT may not only reduce the total number of LNs but also sterilize mesorectal LNs [86–88], the true rate of unidentified pathologically involved LNs is likely to be higher [83]. Adverse prognostic features, including a greater depth of perirectal fat invasion, poor tumor differentiation, the presence of lymphovascular invasion, abnormally elevated pretreatment carcinoembryonic antigen levels (>5 ng/mL), circumferential margin involvement, and a low-lying position may identify T3N0 patients at high risk for local recurrence who may benefit from the addition of radiation therapy. The pretherapy detection of unfavourable features would lead to the delivery of adjuvant CRT. It is unlikely that a pretherapy biopsy would reliably exclude unfavourable pathologic features [83]. An alternative method for identifying positive nodes would be the analysis of molecular markers which is very limited nowadays [83]—for example, tumor location, P21, CD44v6 [89]. On the other hand, neoadjuvant CRT could be a likely overtreatment in the subgroup of T3N0 tumors with favourable features (low risk of CRM involvement and location in the mid/upper rectum). Accurate pretreatment identification of node-negative cancer and subdivision of cT3N0 tumors into different substages are fundamental requirements in evaluating the efficacy and safety of tailored treatments [84]. 

Some institutions consider neoadjuvant short-course RT a valid alternative in patients with cT3 rectal cancer whose disease does not need downsizing (not threatened by CRM, located in the upper and mild rectum), also because of the possibility of choosing the proper adjuvant CT on the basis of pathological data [90]. 

Accurate pretreatment identification of node-negative cancer and subdivision of cT3N0 tumors into different substages are fundamental requirements in evaluating the efficacy and safety of tailored treatments [84]. Thus, neoadjuvant CRT shoud remain the care standard for locally advanced rectal cancer, including cT3N0 on the grounds of the principle that overtreatment is less hazardous than undertreatment for cT3N0 rectal cancers (5) [84]. The optimal treatment for these truly superficial uT3 lesions warrants further study in the form of a randomized trial.

## 8. Quality of Life and Treatment Toxicity

The adverse effects after treatment for rectal cancer include gastrointestinal disorders, genitourinary and sexual dysfunction, and secondary cancers, pelvic or hip fractures, and thromboembolic diseases [91–95]. 

### 8.1. Gastrointestinal Disorders

The symptoms resulting from adverse effects of the gastrointestinal tract include diarrhea, bleeding, abdominal pain and obstruction due to stenosis or adhesions and more rarely malabsorption [96], necrosis, perforation, and fistulation [97]. The incidence of small bowel obstruction requiring surgery following adjuvant pelvic RT for rectal cancer is 4–15% in historical series [67]. Anal and rectal dysfunction refers mainly to symptoms such as gas, liquids or solid faeces incontinence, rectal emptying problems, frequent bowel movements, and diarrhea. The long-term bowel function is impaired more by adjuvant RT than by neoadjuvant RT [5]. Patients with stomas were more satisfied with their bowel function than those operated with a low anterior resection without stoma; those were independent of RT [20, 92]. In a Polish trial comparing neoadjuvant short-course RT and CRT, no differences were seen in the proportion of patients having incontinence to loose stools (72% RT and 65% CRT), difficulties in discrimination between gas and stools (59% RT and 66% CRT) and in stool frequency (median 4 RT and 5 CRT). This study did not reveal any differences in late adverse effects from the gastrointestinal tract [13]. Other gastrointestinal disorders include fistulas and anastomotic strictures. Adjuvant CRT increased the risk of late anastomotic strictures (12%) compared to neoadjuvant CRT (4%, *P* = 0.003) [5].

### 8.2. Genitourinary Dysfunction

Urogenital dysfunction after rectal cancer treatment is common. It include, incontinence, retention, dysuria, frequency and urgency [98, 99]. Late urinary tract symptoms were reported in 4% of all patients in the Western Norwegian trial [100] and in 3% of all patients in the Uppsala trial, with chronic cystitis as the most common diagnosis [101]. Bladder problems were seen in 2% of the preoperatively treated and 4% of the postoperatively treated patients (*P* = 0.21) in the German study on pre- versus postoperative CRT [5].

### 8.3. Sexual Dysfunction

As with surgery, RT can lead to increased sexual dysfunction. In males, a long-term deterioration of ejaculatory and erectile function is due to late radiation damage to the seminal vesicles and small vessels, respectively. In females, RT leads to vaginal dryness and diminished sexual satisfaction [102]. Patients who undergo an APR have more voiding difficulties, erectile dysfunction, and dyspareunia, compared with those who undergo a LAR [102]. Dutch TME trial analysed the grade of sexual dysfunction between irradiated and surgery only patients [102]. In males, the sexual activities of those who were still active preoperatively decreased to 67% in irradiated patients and 76% in nonirradiated patients: this difference was not statistically significant. A greater difference was seen in females with a reduction to 72% for irradiated patients and 90% nonirradiated patients.

### 8.4. Second Cancers

The risk was mainly related to second cancers from organs within or adjacent to the irradiated target. There was no individual type of cancer that could be related to the RT, but gynaecologic and prostate cancers were the most common second cancers from organs within or adjacent to the irradiated target [94].

Gender differences in quality of life of patients after treatment for rectal cancer have been previously reported. Women have higher rates for fatigue and insomnia as shown in general population. Men appear to have higher scores of sexual problems, but somewhat higher scores for sexual functioning than female [102]. The multicenter prospective observational trial [103] evaluating quality of life in patients with rectal cancer who receive neoadjuvant CRT. Only 14% of patients had optimal continence. Physical/social functioning, fatigue, and body image showed a decrease just after neoadjuvant CRT and returned to baseline levels at 1 year after treatment. Global quality of life was stable over time. Male sexual problems were greatly impaired throughout the study period (*P* < 0.001) with major clinically meaningful changes between baseline and 1 year after treatment.

In conclusion, neoadjuvant/adjuvant treatment for locally advanced rectal cancer can have some negative effects on quality of life that should be discussed with the patient before the definitive choice of treatment.

## 9. Risk Factors Associated with Local Recurrence

Due to differences in the lymphatic drainage and the narrow anatomic space of the true pelvis, rectal cancer behaves differently from colon cancer, particularly with regard to an increased risk for local recurrence [104, 105]. Important prognostic factors following complete tumor excision include the lack of distant metastases (M stage), the depth of infiltration into the rectal wall (T stage), the number and localization of involved lymph nodes (N stage), the circumferential radial margin (CRM), and a positive distal margin [106]. Pathologic findings like lymphovascular invasion and poor differentiation have also been shown to increase the risk of local recurrence [107, 108]. A positive microscopic margin is defined as histological evidence of tumor in the line of resection and results in local recurrence rates ranging from 31 to 55% [109–111]. Some authors have advocated that a CRM of 1 or 2 mm should be viewed as a positive margin because high local recurrence are reported with a 0.1 to 1 mm margin (7–28%) and with a 1.1 to 2 mm margin (5–15%) [109–111]. For distal rectal cancer, a clear distal margin of 1 cm is thought to be an oncologically adequate resection in patients who received neoadjuvant CRT [112]. Finally, patients who undergo APR tend to have a higher positive CRM rate than those who undergo SSS [113]. Local excision was also associated with increased local recurrence rates, particularly when excision was performed for more advanced-stage tumors (such as any tumor greater than a T1) or cancers with poor prognostic pathologic factors (such as neurovascular invasion). Leibold et al. examined the prognostic significance of the location of involved lymph nodes in 121 rectal cancer patients (uT3-4 and/or N+) following neoadjuvant CRT [114]. The data suggested that, following neoadjuvant CRT, proximal lymph node involvement (those lymph nodes along the major supplying vessels, in contradistinction to the mesorectal lymph nodes) is associated with a high incidence of metastatic disease at time of surgery. 

Other prognostic parameters (e.g., newer molecular markers, genetic signatures, etc.) are of interest but cannot yet be regarded as a routine part of clinical decision making.

## 10. Issues for the Future

Unfortunately, neoadjuvant CRT is not beneficial for all patients. The treatment response ranges from a pCR to a resistance. It is reported that 10 to 20 percent of patients with advanced rectal cancer show pCR to neoadjuvant CRT [58, 59]. In addition, complete or near-complete response to neoadjuvant CRT is indicative of improved long-term prognosis [59, 115]. Nowadays, it is not possible to identify patients with no or minimum tumor response to neoadjuvant CRT before its initiation. Based on our data, group of nonresponders represents approximately 12% of all patients [116]. Although several molecular markers have been investigated as potential predictors of therapeutic responses, no marker has been consistently identified as clinically applicable. In the future, molecular predictors and improved imaging could be used to individualize the therapy of patients with locally advanced rectal adenocarcinomas, and thus in a selected group of nonresponders avoid the delay of surgical intervention and the eventual toxicity of the neoadjuvant therapy. Integration of targeted therapies, newer cytotoxic agents, and a more selective approach to the use of adjuvant chemo-targeted therapy strategies should be defined.

## Abbreviations

MRI: magnetic resonance imaging
EUS: endoscopic transrectal ultrasound
APR: abdominoperineal resection
SSS: sphincter saving surgery
TME: total mesorectal exsision
5-FU: 5-fluorouracil
RT: radiotherapy
CT: chemotherapy
CRT: chemoradiotherapy
pCR: pathologic complete response
ypT: posttreatment T stage
ypN: posttreatment N stage
TRG: tumor regression grade
EGFR: epidermal growth factor receptor
VEGF: vascular endothelial growth factor
sVEGFR1: soluble vascular endothelial growth factor receptor
PlGF: placental-derived growth factor
CEC: circulating endothelial cell
DFS: disease-free survival
OS: overall survival
CRM: circumferential radial margin
NCCN: National Comprehensive Cancer Network
Gy: Gray
LN: lymph node
HFS: hand-and-foot syndrome lymph node.
